# 304. Impact of Antibiotic Prophylaxis Prior to Treatment with Steroids and Tocilizumab in COVID-19 Patients

**DOI:** 10.1093/ofid/ofab466.506

**Published:** 2021-12-04

**Authors:** Francisco Javier Membrillo de Novales, Miriam Estébanez Muñoz, Tatiana Mata Forte, Germán Ramírez-Olivencia, María Isabel Zamora Cintas, María Simón Sacristán, María Sánchez de Castro, Carlos Gutiérrez Ortega, Lucía Elena Ballester Orcal

**Affiliations:** Hospital Central de la Defensa “Gómez Ulla”, Madrid, Madrid, Spain

## Abstract

**Background:**

The incidence of bacterial or fungal coinfections in COVID-19 patients is low. The incidence of nosocomial superinfections is higher, especially related to ICU admission. Treating COVID-19 with steroids plus tocilizumab (TCZ) has been associated with superinfections. Therefore, the use of antibiotic prophylaxis prior to infusion of TCZ could be considered to reduce the risk of life-threatening superinfections in critically ill patients.

**Methods:**

Retrospective, single center cohort study. COVID-19 patients older than 14 years, admitted to Hospital Central de la Defensa (Madrid, Spain) from Mar 5th to Nov 24th, 2020 with a diagnosis of COVID-19 were included. Local protocols suggested antimicrobial prophylaxis before the infusion of TCZ. Medical records, treatments received, and microbiological data of all patients who received TCZ were reviewed. Microbiological isolates were considered in the 14 days following the administration of TCZ. Two ID specialists independently reviewed the medical record and decided to qualify the isolate as superinfection or colonization.

**Results:**

2,069 patient records were analyzed. 70 patients received TCZ; all of them were admitted to ID wards and under steroid treatment. 45 (64,5%) patients received antibiotic prophylaxis. The preferred antibiotics were ceftriaxone (N = 18) and ceftobiprole (N = 14). No significant differences were found in age, Charlson index or COVID-19 SEIMC-Score. 24 isolates were detected in 14 patients (18 bacterial, 6 fungal). 17 isolates were considered superinfections; the most frequent isolates were *C. albicans* (N=5), *E. faecalis* (N=3) and *S. epidermidis* (N=2). There were no statistically significant differences between the different prophylaxis strategies in terms of in-hospital mortality or ICU admission. However, patients who received ceftobiprole tended to have fewer isolates and fewer superinfections than those receiving ceftriaxone (ceftobiprole group: 2 isolates in 1 patient, 1 (7,1%) patient with superinfection; ceftriaxone 11 isolates in 5 patients, 4 (22,2%) patients with superinfection) (*p*= 0,35, Fisher exact test).

Table 1. Characteristics of study population.

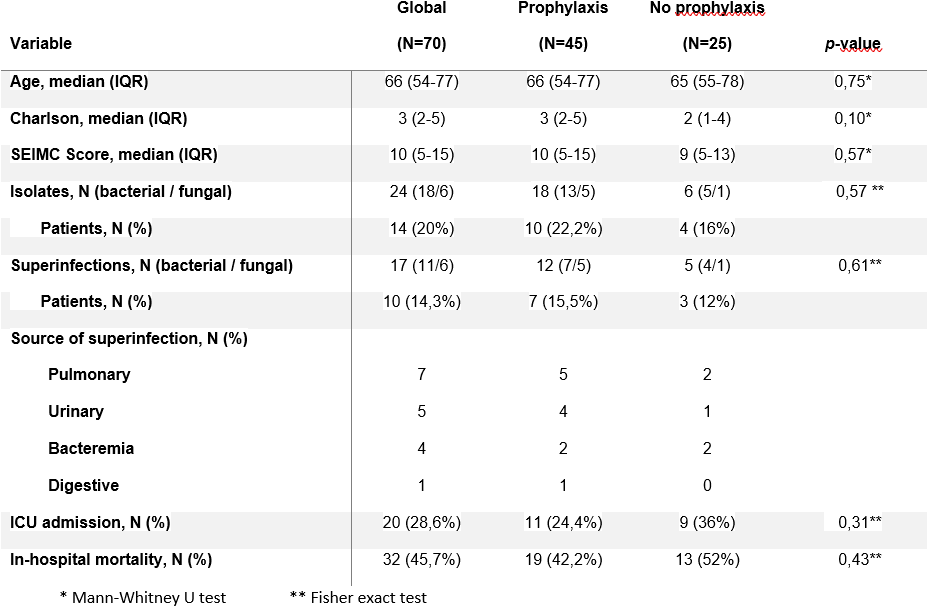

Table 2. Outcomes according to antimicrobial prophylaxis prior to Tocilizumab.

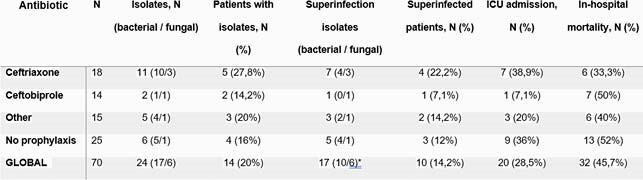

Table 3. Description of isolates.

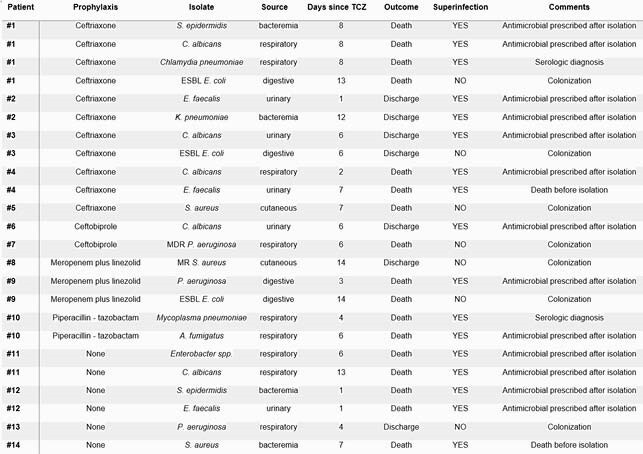

**Conclusion:**

Antibiotic prophylaxis prior to infusion of TCZ in patients with COVID-19 and receiving steroids could determine the profile of bacterial and fungal superinfections.

**Disclosures:**

**All Authors**: No reported disclosures

